# The effect of zinc on healing of renal damage in rats

**DOI:** 10.15171/jnp.2017.27

**Published:** 2017-02-03

**Authors:** Mehdi Salehipour, Ahmad Monabbati, Mohammad Reza Ensafdaran, Ali Adib, Amir Hossein Babaei

**Affiliations:** ^1^Department of Urology, Shiraz University of Medical Sciences, Shiraz, Iran; ^2^Department of Pathology, Shiraz University of Medical Sciences, Shiraz, Iran; ^3^Student Research Committee, Shiraz University of Medical Sciences, Shiraz, Iran

**Keywords:** Kidney, Trauma, Wound Healing, Zinc

## Abstract

**Background:**

Several studies have previously been performed to promote kidney healing after injuries. Objectives: The aim of this study was to investigate the effect of zinc on renal healing after traumatic injury in rats.

**Materials and Methods:**

Forty healthy female rats were selected and one of their kidneys was incised. Half of the incisions were limited only to the cortex (renal injury type I) and the other ones reached the pelvocalyceal system of the kidney (renal injury type II). All the rats in the zinc treated group (case group) received 36.3 mg zinc sulfate (contained 8.25 mg zinc) orally. After 28 days, the damaged kidneys were removed for histopathological studies.

**Results:**

In the rats with type I injury, kidney inflammation of the case group was significantly lower than that of the control group. However, the result was not significant in rats with type II injury. Tissue loss and granulation tissue formation were significantly lower in the case group than the control group in both type I and II kidney injuries.

**Conclusions:**

Overall, Zinc can contribute to better healing of the rat’s kidneys after a traumatic injury.

**Implication for health policy/practice/research/medical education:**

Zinc is an essential micronutrient with several functions in body. It is proven that zinc has important roles in wound healing mechanisms. Results of this study indicate that zinc can accelerate the process of renal healing after kidney laceration in rats. Kidney damage occurs through renal surgeries and penetrating trauma. Therefore, zinc can be added to treatments after renal surgeries to help better healing and reducing admission course in hospital.

## 1. Introduction

Wound healing is a biological survival mechanism which starts immediately after destruction of the tissue or skin integrity. This process consists of three main phases: inflammation, proliferation and tissue remodeling (1). Fibroplasia, angiogenesis and re-epithelialization occur in proliferation phase which results in extracellular matrix and granulation tissue formation (2). Hypertrophic scar tissue or keloids are the result of imbalance of synthesis and degradation of matrix formation in remodeling phase which leads to abnormal collagen deposition (3). Various factors including adhesion molecules, growth factors, proteinases, and cytokines have been found to be involved in the process of wound healing (2). Previous studies showed that some mechanical factors can led to better tissue healing. For example, gentle tissue handling, the use of thin and absorbable suture thread, and a tension-free closure (4).

Kidney is a vital and also vulnerable organ. Physiologic fluid balance and electrolyte stability heavily relies on the proper functioning of the kidney. Therefore, renal injury should be rapidly treated in order to prevent its complications. Kidney injury occurs iatrogenically in most renal surgical procedures like open nephrolithiotomy, percutaneous nephrolithiotomy, partial nephrectomy, retrograde renal surgery, and kidney biopsy. Furthermore, several mechanisms and substances may lead to renal injury, including inflammation, autoimmune reaction, reactive oxygen spices, oxidative damage, drugs, and physical trauma (5).

Kidney is an organ with little potency for self-regeneration (6) as compared to some other organs like the skin. This is probably attributed to low number of stem cells in the renal tissue while numerous populations of stem cells are in all the skin layers (7). The administration of exogenous stem cells results in better healing of the renal tissue (8).

The important effect of nutrition on wound healing was found many years ago (9). It is known that a good healing process demands both macronutrients and micronutrients (9,10). For example, some vitamins like vitamins A or C accelerate the process of wound healing (11). Also, minerals are the other type of micronutrients that influence wound healing. Zinc is an important micronutrient that plays several physiological roles in the body. It is required for activating growth (12). Zinc fights against infection and has the strongest effect on the immune system as compared to all other vitamins and minerals (13). Zinc has been proven to have accelerating effect on wound healing by several mechanisms (14). Based on our search, there is no study investigating the effect of zinc on healing of traumatic renal injury.

## 2. Objectives

The aim of this experimental study was to investigate the effect of zinc on renal wound healing.

## 3. Materials and Methods

This study was conducted in the animal laboratories affiliated with Shiraz University of Medical Sciences, Shiraz, Iran.

Forty healthy female rats weighting 200 ± 20 g and aged 10 to 12 weeks were closely examined. None of the rats was used in previous experiments. If any of the rats died during the first week of the experiment, it was excluded from the study. Also, infection within the experiment course was another exclusion criterion. The rats were all at the initiation of their menstrual cycle. They were randomly divided into two groups, the zinc treated group (case group) and the control group. In each of case and control groups, half of the rats had only renal cortex injury (type I), and the rest of them had cortex and pelvocalyceal system damage (type II). Therefore, the rats were divided into four groups in this study, case group with type I renal injury, case group with type II renal injury, control group with type I renal injury, and control group with type II renal injury. Each group was composed of ten rats.

Rats’ tails were numbered and labeled from 1 to 40. At first, 20 rats were selected from all 40 rats. These rats were assigned to type I injury group. Again, random selection of cases was performed on these 20 rats and 10 rats were selected and labeled as type I injury case group. Other 10 rats in type I injury group were labeled as control group. All 20 rats that were not selected in the first stage of randomization, were assigned to type II injury group. The same randomization method was done on rats with type II injury and they were equally divided to case and control groups.

In the first step, anesthesia was induced for all the included rats by injecting a mixture of ketamine and xylazine by the dose of 100 mg/kg and 8 mg/kg, respectively. Then in an aseptic procedure, through a midline abdominal incision, left colon was mobilized medially, left Gerota’s fascia was dissected, and left kidney was explored. Then an incision was made vertically in cortical part of left kidney (in type I) and extended and bypassed corticomedullary junction (in type II). If the cuts were limited to the renal cortex, they were regarded as type I injury and if they reached the pelvocalyceal system, as a more severe injury, they were regarded as type II renal injury. To prevent bleeding, the site of incision was approximated by chromic 3-0 suture. After placing the damaged kidney in its anatomic position, skin incision was sutured. Then, the rats were transferred to an aseptic cage after recovering from anesthesia. Both groups received rodent food and water. Only the case group received oral zinc on a daily basis 36.3 mg zinc sulfate (contained 8.25 mg zinc). No other supplements or drugs were given to the animals. The rats’ health status was observed for any signs of infection.

After 28 days, anesthesia was induced for all the rats with the same technique and their left nephrectomy was performed. Then renal tissue samples were immediately collected for histopathological studies. The organ tissue was fixed in 10% buffered formalin solution within room temperature. After 24 hours, paraffin embedding was done and 5 μm slides prepared. Then the slides were stained with hematoxylin and eosin (H & E) and then evaluated for histopathological changes.

A pathologist reviewed the kidney both macroscopically and microscopically. Presence of inflammation was checked based on the existence of inflammatory blood cells in the tissue. Less than 10 WBC/HPF (white blood cell/high power field) was considered as no inflammation. Neutrophils 10-100/HPF was regarded as mild inflammation and after that considered as moderate to severe inflammation. The amount of granulation tissue and calcification was recorded. Dystrophic calcification was reported as either present or absent. The maximum width of the tissue loss and granulation tissue was measured by mm. This was a rough estimation of the tissue loss. The pathologist did not have any information about the grouping methods and was completely blind toward the study.

### 3.1. Ethical issues

The research followed the tenets of the Declaration of Helsinki. The research was approved by ethical committee of Shiraz University of Medical Sciences. Prior to the experiment, the protocols were confirmed to be in accor­dance with the guidelines of Animal Ethics Committee of Shiraz University of Medical Sciences. All procedures were performed under general anesthesia in order to respect for the animals’ rights.

### 3.2. Statistical analysis

After recording the data, it was sent to a biostatistician for proper statistical analyses. Also the biostatistician was blind toward this study. Random allocation of the rats and group differences analysis were done using SPSS (IBM SPSS, version 13). Inflammation, granulation tissue formation, calcification, and tissue loss were compared between the case and control groups using chi-square test. Differences of age and sex of the case and control groups were analyzed using independent *t* test after assuming the equality of variances by Levene’s test. *P* value under 0.05 was considered as statistically significant.

formation and calcification in the case group, control group and total according to pathological studies

## 4. Results

There was no significant difference between the two groups regarding sex and weight.

Presence or absence of inflammation, granulation tissue, calcification, and the amount of tissue loss are shown in Table 1.

**Table 1:** Evaluation of zinc effect in improvement of kidney tissue repair after trauma in rat, inflammation, tissue loss, granulation tissue formation and calcification in the case group, control group and total according to pathological studies

**Measured parameters**		** Type I injury (20) **			** Type II injury (20) **			**Total**		
		** Case (10) **	** Control (10) **	*P*	** Case (10) **	** Control (10) **	*P*	** Case (20) **	** Control (20) **	*P*
Inflammation	No inflammation	5	0	0.016^a^	1	0	0.500^a^	6	0	0.010^a^
	Mild to moderate	5	10		9	10		14	20	
Tissue loss	No tissue loss	10	3	0.001^b^	5	0	0.00^b^	15	3	<0.001^b^
	1–3 mm	0	4		1	0		1	4	
	More than 3 mm	0	3		4	10		‏4	13	
Granulation tissue formation	Yes	0	10	<0.001^b^	5	10	0.016^b^	5	20	<0.001^b^
	No	‏10	‏0		‏5	‏0		‏15	‏0	
Calcification	Yes	0	2	0.152^b^	1	4	0.237^b^	1	6	0.046^b^
	No	10	8		9	6		19	14	

^a^Fisher’s exact test. ^b^Fisher’s exact test (1-sided).

In rats with type I injury, five rats from the case group had no inflammation but five of them had mild to moderate inflammation, while all the rats in the control group had mild to moderate inflammation which was significantly higher than the case group.

In rats with type II injury, one rat from the case group had no inflammation but nine of them had mild to moderate inflammation. All rats from the control group had mild to moderate inflammation. This difference was not statistically significant.

Overall, six rats in the zinc treated group had no inflammation but 14 of them had mild to moderate inflammation but all rats in the control group had mild to moderate inflammation that was significantly higher than that of the case group.

In type I injury, there were no tissue losses in all the case group. But in the control group, 4 rats had 1-3 mm and 3 had more than 3 mm tissue loss, which was statistically significant compared with zinc treated rats.

**Figure 1:**
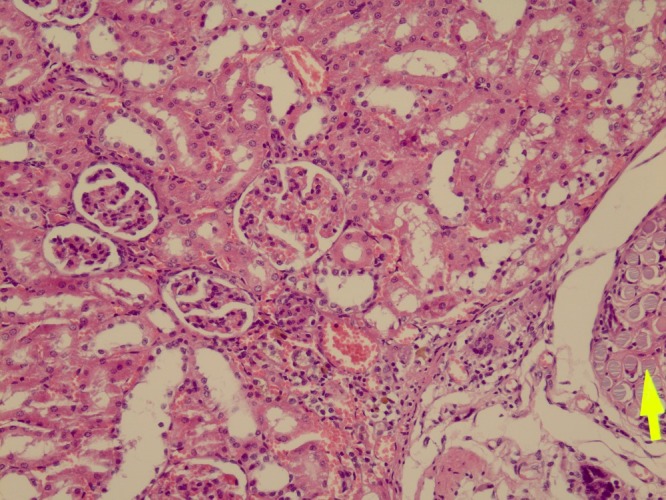
This figure shows high power view of near complete healing of injury with no significant tissue loss in the case group. Suture material is seen in the right side, (arrow) (H&E ×400).

In type II injury, five rats of the case group had no tissue loss; one had 1-3 mm and 4 had more than three mm tissue loss. All the rats in the control group had more than three mm tissue loss. The difference was statistically significant compared to the zinc treated group. Complete healing without loss of tissue in the zinc treated group is shown in Figure 1.

Granulation tissue formation was significantly lower in the case groups than the control groups in both type I and II injury (Table 1).

**Figure 2:**
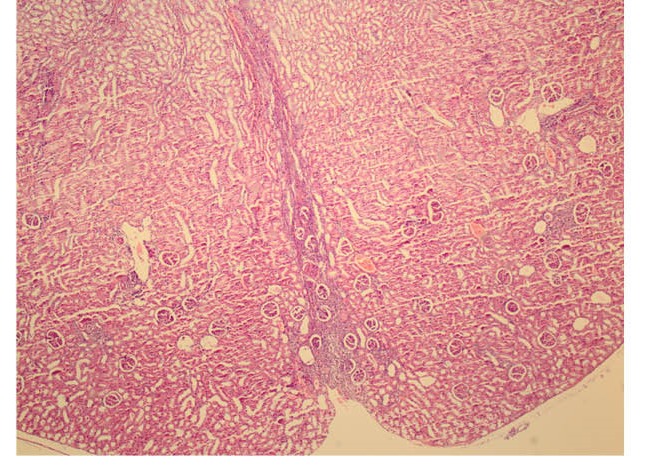
A line of healing with narrow band of granulation tissue is seen in a full thickness injury which involves calyces in the case group( H&E ×100).

**Figure 3:**
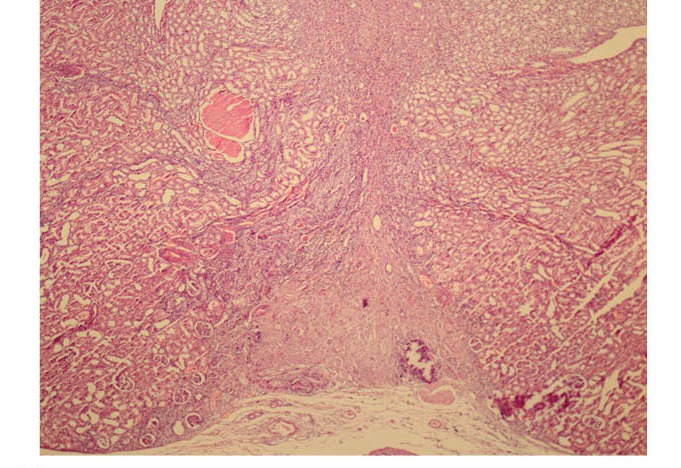
This image is a low power view of the renal cortex which indicates tissue loss and granulation tissue formation in the control group (H&E ×100).

Granulation tissue formation of the case and control groups are demonstrated in Figures 2 and 3, respectively.

Overall, calcification was significantly lower in the case group than the control, but there was no significant difference between the case and control groups regarding the degree of trauma (Table 1).

## 5. Discussion

The results of this study show that administration of zinc can significantly decrease inflammation in rats with type I kidney injury. Granulation tissue formation and tissue loss decreased in both type I and II cases. Zinc administration did not significantly decrease the kidney tissue calcification in the case group in comparison with the control group with regards to the degree of trauma.

Most of the previous studies were performed to promote kidney healing after non-traumatic injuries (15).

Jacquillet et al investigated the effect of chronic Cadmium chloride (CdCl_2_) intoxication in rats. They found out that treating rats with zinc protects their kidney against the toxic effects of Cd^2+^ by preventing altered claudin expression and inhibiting apoptosis (16).

In a study by Kheradpezhouh et al, the effect of curcumin and combination of curcumin and N-acetyl cysteine (NAC) was investigated on acetaminophen induced hepatorenal toxicity. They found out that curcumin can protect the liver and kidney from the acetaminophen toxicity. Also, curcumin in combination with NAC, significantly decreased the therapeutic dose of NAC (17).

Another research investigated the effect of montelukast as a cysteinyl-leukotriene receptor antagonist on gentamicin-induced nephrotoxicity and oxidative damage in rat kidney. They showed that montelukast can prevent kidney damage by its antioxidant effect (18).

Salehipour et al evaluated the effect of parenteral vitamin E on renal ischemia-reperfusion injury in rabbits’ kidney. Histopathological evaluation of the kidneys revealed that parenteral injection of vitamin E can significantly protect the renal cells against ischemia-reperfusion injury (19).

Although numerous researches were done to investigate healing process after non-traumatic kidney injuries, according to our search, no article was found that had surveyed the effect of micronutrients on the healing process of kidney wounds.

In some studies, researchers found that similar healing processes occurred after traumatic and non-traumatic kidney injuries (20,21).

The role of zinc in wound healing is proven. Previous studies showed that zinc can increase the rate of healing in pressure ulcers (22), re-epithelialization of the wounds (23), the mitotic index of the epidermal basal cells, and the strength of abdominal skin incisions in rats (24).

In these studies, evidence for the functional role of zinc in repairing tissues was explained by anti-oxidant effect, anti-microbial effect and the function of zinc metalloenzymes, such as zinc finger proteins and matrix metalloproteinases (MMPs) (24).

Zinc finger proteins have an important role in regulating gene expression (25). They regulate genes which are responsible for production of growth factors in wound healing (26).

Extracellular zinc mimics growth factors and activates intracellular mitogen-activated protein kinase (MAP kinase) and protein tyrosine phosphorylation. Zinc, in coordination with calcium, promotes the fibroblast growth during wound healing (27).

MMPs, an essential zinc-dependent endopeptidase, play an important role in tissue remodeling (28). Zinc-dependent MMPs are responsible for auto-debridement and keratinocyte migration in wound healing. They also contribute to wound contraction (14).

Zinc protects the cells against reactive oxygen species (ROS) and bacterial toxins by the zinc finger-trans activating protein A. Zinc increases the activation of antioxidant proteins, molecules and enzymes, such as glutathione, catalase, and superoxide dismutase. It also reduces the activities of oxidant-promoting enzymes such as nicotinamide adenine dinucleotide phosphate (NADPH) and inhibits the lipid peroxidation products. The plasma zinc concentrations can inversely correlate with the changes in concentration of high-sensitivity C-reactive protein (hsCRP) after zinc supplementation (29).

Also, zinc inhibits the activation of nuclear factor-kappa B (NF-kB) by preventing the induction of messenger RNA of interleukin 1, beta (IL-1b) and tumor necrosis factor-alfa in mononuclear cells (30).

Zinc inhibits the bacterial growth when it reaches the super-physiological level in topical administration (24).

Our results demonstrated the effectiveness of zinc in treatment of traumatic kidney injuries, particularly in type I injury. This difference may be due to severity of injury. The amount of calcification was not significantly different in the case and control groups. It may be due to the fact that calcification has fewer roles, in proportion to other measured parameters, in determining the progress of kidney healing.

There were a few limitations in our study. The best animal for studying kidney injuries is pig. Hence, if we had conducted our study on the pig model instead of rat, the results would be more extensible to human.

The second important limitation was the absence of serum zinc measurements before and after treatment.

## 6. Conclusions

In conclusion, zinc can reduce inflammation, granulation tissue formation, and tissue loss of kidney wound in rats. Further studies are required in order to fully investigate the role of zinc in kidney wound healing.

## Acknowledgements

The authors would like to thank Center for Development of Clinical Research of Nemazee Hospital and Dr. Nasrin Shokrpour for editorial assistance.

## Authors’ contribution

Study concept and design was done by MS and AM. Acquisition of data, analysis, and drafting of the manuscript was done by MRE, AA, and AHB. All authors read and signed the final paper.

## Conflicts of interest

The authors declare no conflicts of interest.

## Funding/Support

This article is extracted from urology residential thesis of Mohammad Reza Ensafdaran (Thesis #91-01-01-4493).
